# Fully Convolutional DenseNet with Multiscale Context for Automated Breast Tumor Segmentation

**DOI:** 10.1155/2019/8415485

**Published:** 2019-01-14

**Authors:** Jinjin Hai, Kai Qiao, Jian Chen, Hongna Tan, Jingbo Xu, Lei Zeng, Dapeng Shi, Bin Yan

**Affiliations:** ^1^National Digital Switching System Engineering and Technological Research Center, Zhengzhou, Henan Province, China; ^2^Department of Radiology, Henan Provincial People's Hospital, Zhengzhou, Henan Province, China

## Abstract

Breast tumor segmentation plays a crucial role in subsequent disease diagnosis, and most algorithms need interactive prior to firstly locate tumors and perform segmentation based on tumor-centric candidates. In this paper, we propose a fully convolutional network to achieve automatic segmentation of breast tumor in an end-to-end manner. Considering the diversity of shape and size for malignant tumors in the digital mammograms, we introduce multiscale image information into the fully convolutional dense network architecture to improve the segmentation precision. Multiple sampling rates of atrous convolution are concatenated to acquire different field-of-views of image features without adding additional number of parameters to avoid over fitting. Weighted loss function is also employed during training according to the proportion of the tumor pixels in the entire image, in order to weaken unbalanced classes problem. Qualitative and quantitative comparisons demonstrate that the proposed algorithm can achieve automatic tumor segmentation and has high segmentation precision for various size and shapes of tumor images without preprocessing and postprocessing.

## 1. Introduction

Breast cancer is the most common disease of women and has become the second disease which leads to death [1]. The number of breast cancer patients is increasing gradually since 1970s. Early detection of breast cancer is beneficial for improving the survival rate and survival quality. Mammography is the most conventional and noninvasive examination and is an effective screening method for early detection and diagnosis of breast cancer. Tumor segmentation provides morphological features and is an essential step for tumor analysis and classification.

Traditional studies on tumor segmentation mainly rely on gray-level and texture features which are distinct to partition mammogram into different regions. Region-based methods start from a set of manually located seed points or small patches containing suspicious region, such as region growing [2] and watershed methods [3]. Different location of seed points are set to segment tumor according to different image preprocessing methods, such as Gaussian filtering [4, 5] and mathematical morphological operation [6]. Watershed methods mainly used some preprocessing algorithms to reduce the number of initial segmented basins [7, 8]. Active contour model, especially level set [9] is also used for breast masses segmentation. A radial gradient index- (RGI-) based segmentation method is applied to yield an initial contour closer to the lesion boundary location [10]. 3D radial-gradient index segmentation and 3D level set-based active contour algorithm [11] are also applied to 3D CT breast images. A feature embedded vector-valued contour-based level set method [12] is proposed to perform mammographic mass segmentation. It used level set method to obtain the initial boundaries on the smoothed mammogram as the shape constraint to design stopping function and integrated texture maps, gradient maps, and the original intensity map to obtain more accurate segmentation.

Region-based and contour-based methods are all unsupervised. There are also some supervised segmentation algorithms based on deep networks. Dhungel et al. [13] combined multiple deep belief networks (DBNs), Gaussian mixture model (GMM) classifier, and a prior of location, size, and shape of the mass as potential functions and used structured SVM to learn a structured output and perform segmentation. They further used conditional random field (CRF) with tree reweighted belief propagation as structured prediction function to boost the segmentation performance [14]. The output of convolutional neural network (CNN) is also introduced as a complementary potential function in addition to the aforementioned potential functions, yielding state-of-the-art segmentation performance [15]. These methods all used two-stage training. An end-to-end network was proposed based on the mass region of interest (ROI) images [16]. It employed fully convolutional network to model potential function, followed by a CRF to perform structured learning, and integrated adversarial training to learn robustly from scarce mammographic images.

CNN has powerful ability of extracting abstracted features directly from the raw input data and achieves remarkable achievements in computer vision fields, such as image classification [17–19], object detection [20–22], and image segmentation [23–25]. Image segmentation methods based on CNN could get segmentation result through discriminating every pixel in the image. Long et al. [23] proposed the fully convolutional network (FCN) and performed fine tuning in an end-to-end manner based on pretrained VGG-Net [26] for image semantic segmentation. FCN replaced the fully connected layers with the convolutional layers in order to keep the location information, which adjusted the classification network for the segmentation task. Additionally, FCN employed the skip architecture to merge semantic features and detailed features and performed deconvolution to obtain more accurate segmentation results. Since that, the deconvolution operation had been widely used in many semantic segmentation networks. Hyeonwoo [24] proposed the symmetrical encoder-decoder architecture called DeconvNet. The DeconvNet employed successive unpooling layers which reconstruct the original size of activations through recording the locations of maximum activations selected during pooling operation and deconvolution layer with learned filters to generate dense pixelwise class probability map. SegNet [25] is another similar symmetrical network which used convolution after unpooling layer to refine the sparse feature maps. Due to the low localization precision of reconstructing the resolution directly from high-level features, U-Net [27] combined the upsampling output with the high-resolution features from the encoding path to improve the segmentation performance. Simon et al. [28] extended DenseNet [29] which achieved excellent results on image classification tasks to deal with the problem of semantic segmentation. The proposed Fully Convolutional DenseNet (FC-DenseNet) also employed skip connections that the feature maps from the downsampling path are concatenated with the corresponding feature maps in the upsampling path and achieved state-of-art results. In conclusion, the typical segmentation architecture built on CNN is mostly fully convolutional network with encoder-decoder architecture training in an end to end manner. It mainly composes of a downsampling path responsible for extracting coarse semantic features and an upsampling path trained to recover the input image resolution.

Because of the larger size of digital mammograms, the segmentation time of active contour model, such as level set, is greatly increased when the whole image is automatically segmented. And most of traditional unsupervised methods rely on low-level features such as the image gray value, texture, gradient, and other information or the initial priori settings, such as the initial seed points of region growing and initial contour of level set method. But this cannot achieve fully automatic segmentation of breast tumor, and segmentation precision is influenced by the hand-crafted features and initial priori position. In addition, some image backgrounds are complex and similar to the characteristic of tumor region, such as the pectoral muscles, or the gray value of internal and external region of the tumor have small differences. So, many aforementioned segmentation methods are based on the tumor-centric candidate box [30, 31]. Although some supervised segmentation algorithms take into account the category information and can automatically extract tumor features to optimize the segmentation model, they also add priori information of the location, size, and shape of the tumor. And they are also based on the small tumor candidate box and cannot directly segment the entire large size mammograms.

The main goal of our proposed algorithm is to segment the breast tumor on the entire digital mammogram not on the tumor-centric rectangle region which is commonly used in most breast tumor segmentation algorithms. FC-DenseNet further exploited the feature reuse by upsampling the feature maps created by the preceding dense block and used skip connections to help the upsampling path recover spatially detailed information from the downsampling path. It outperforms current state-of-the-art results neither using pretrained parameters nor any further postprocessing. So, we extend FC-DenseNet to achieve automatic tumor segmentation. At the same time, considering that the multiscale information [32, 33] is benefit for improving the segmentation precision of different sizes of tumors, atrous spatial pyramid pooling (ASPP) [34] is added to FC-DenseNet semantic segmentation network. Without significantly increasing the number of learning parameters, ASPP extracts multiscale features by concatenating different sampling rates of atrous convolution [35] to enlarge the receptive field. Loss function is also improved to solve the extremely unbalanced class problem according to the proportion of tumor and background pixels in the entire image. We evaluate this algorithm in our collected digital mammogram dataset, and results demonstrate that our proposed algorithm has yielded better performance than other algorithms.

## 2. Methods

### 2.1. Review of FC-DenseNet

FC-DenseNet [28] is an extension of excellent DenseNet [29] classification network in semantic segmentation by adding an upsampling path to recover the full input resolution. Downsampling path of FC-DenseNet is composed of dense block layer and transition down layer. Dense block layer is composed of batch normalization [36], followed by ReLU [37], a 3 × 3 same convolution (no resolution loss) and dropout with probability *p*=0.2, which is different from DenseNet. A transition down layer is composed of a 1 × 1 convolution (which conserves the number of feature maps) followed by a 2 × 2 pooling operation. Upsampling path consists of dense block layer and transition up layer. Transition up includes a 3 × 3 transposed convolution with stride 2 to compensate for the pooling operation in the transition down. The upsampled feature maps are then concatenated to the ones with the same resolution from the downsampling path to form the input of a new dense block. But in order to prevent the linear growth of feature maps, the input of a dense block is not concatenated with its output. Thus, the transposed convolution is applied only to the feature maps obtained by the last dense block and not to all feature maps concatenated so far. The final layer in the network is a 1 *×* 1 convolution followed by a softmax nonlinearity function to predict the class label at each pixel.

### 2.2. ASPP-FC-DenseNet Segmentation Algorithm

**Figure 1:**
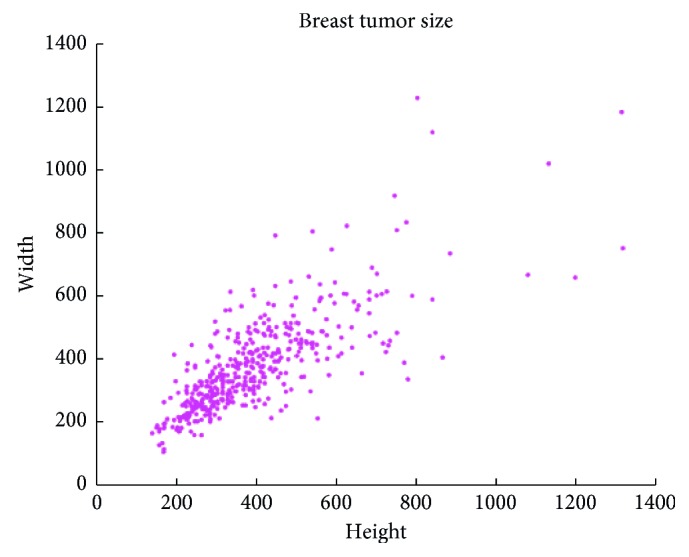
The distribution of breast tumor size.

As the shape and size of malignant tumor are various, as shown in Figure 1, the height and width of the tumor are mostly distributed in 200 and 800 pixel intervals. As the receptive field of single-image scale or small convolution kernel is relatively fixed, it is only effective to present the image features within the scope of the receptive field and cannot be well depicted on the edges of different sizes of tumor. This could influence the segmentation precision. Therefore, extracting multiscale information of the image is helpful to improve the segmentation precision of different sizes of tumors.

Multiscale images (image pyramid) [38] are commonly used as the input of network to extract features for each scale input, and the segmentation results of each scale are linearly interpolated and fused. But all layers of parallel CNN need to compute the features of multiple scale inputs, and the consumption of feature computation is large. Different sizes of convolution kernel have different receptive fields, and exploiting different convolution kernel size [39] to extract multiscale image features is and alternative approach. However, multiple parallel branches with different size of convolution kernels greatly increase the network width and the network learning parameters. Due to the small digital mammogram segmentation datasets, the network is easy to overfit. Therefore, exploiting multiple field-of-views to extract multiscale image features with relatively less increasing parameters is more requisite.

Spatial pyramid pooling (SPP) [40] is a common way to obtain multiscale image information, which was originally proposed to solve the problem of arbitrary input size of proposals in object detection. According to the requirement of the output dimension, SPP divides the input image with arbitrary size into the corresponding number of spatial bins, then the pooling operation is performed on each spatial bin. The features of each spatial bin are combined to get fixed features output dimension. It can be seen that the output of SPP layer fuses the features of different level image bins, thus improves the recognition accuracy. But for image segmentation requiring pixel level semantic classification, multiple pooling operations lose the image details and spatial position information.

**Figure 2:**
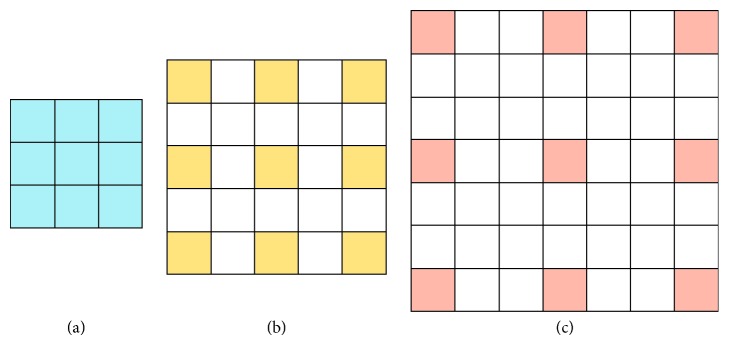
Illustration of atrous convolution with different atrous sampling rates in 1-D. (a) Rate = 1. (b) Rate = 2. (c) Rate = 3.

Atrous convolution [35] is an effective way to expand the network receptive field without increasing the size of convolution kernel and network parameters and is mainly operated by setting different atrous sampling rate. As shown in Figure 2, atrous convolution is performed standard convolution with filter ‘with holes' according to the sampling rates, and the weights of the ‘holes' are reset to 0. Atrous convolution with rate *r* introduces *r* − 1 zeros between consecutive filter values, effectively enlarging the kernel size of a *k* × *k* filter to (*k*+(*k* − 1)(*r* − 1))(*k*+(*k* − 1)(*r* − 1)) filter without increasing the number of parameters. The atrous convolution operation could convolute every pixel of the input by setting a specific convolution stride. By setting the sampling rate, the small convolution kernel can also achieve the effect of large convolution kernel, thus expanding the receptive field of the network without requiring learning any extra parameters and increasing the amount of computation. Atrous convolution could arbitrarily enlarge the field-of-view of filters at any network layer. As shown in Figure 2, the 3 × 3 convolution kernel has the same receptive field of 5 × 5 and 7 × 7 convolution kernel by setting different sampling rates. According to the idea of spatial pyramid pooling, the extracted features of network are resampled using parallel atrous convolutional layers with different sampling rates, and then, the features extracted from each sampling rate are fused to generate the final result. As a consequence, the image features of different sizes of receptive fields are fused to be used to predict the object label, and this approach is called atrous spatial pyramid pooling (ASPP) [34].

**Figure 3:**
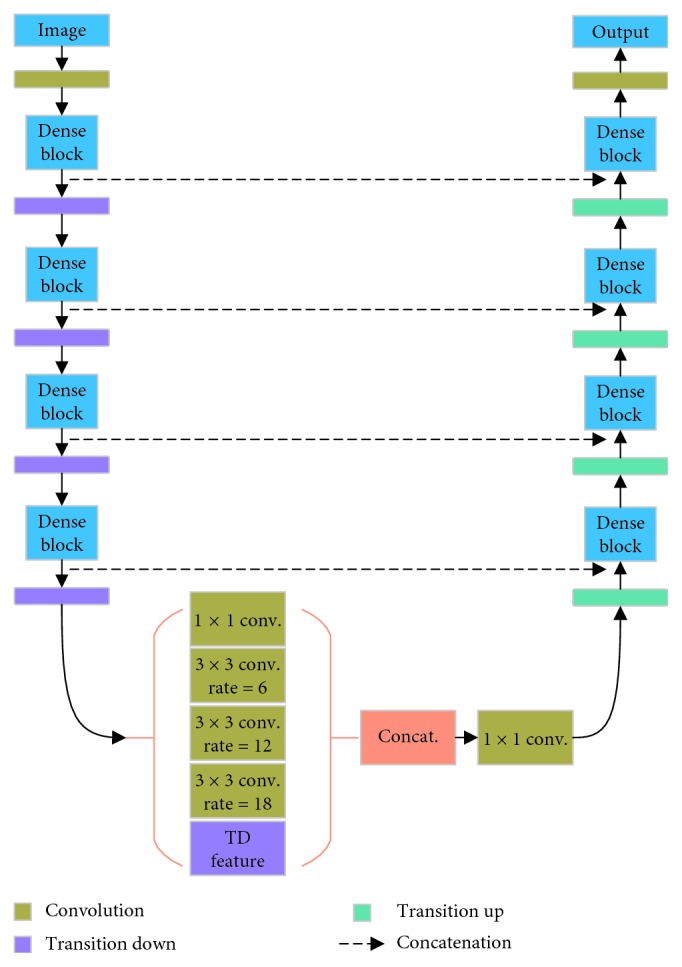
The proposed ASPP-FC-DenseNet.

Because the size of digital mammograms is 4096 × 3328, FC-DenseNet needs many downsampling operations to reduce the resolution of feature map to acquire the abstracted image features, and this increases the learning parameters and is easy to lead network overfit. In addition, it leads to the deep network and the large computation and memory consumption. So, we resized the input image to 512 × 512. For the smaller tumors which have 200 pixels, the final size is almost 30 pixels. But FC-DenseNet has 5 pooling layers, and the down sample rate is 32, resulting in omitting the small size tumor. Although the upsampling path of FC-DenseNet is concatenated with the features extracted from the downsampling path though skip connection, but this still affects the final segmentation precision. Therefore, the downsampling operation of FC-DenseNet is reduced to 4. At the same time, the dense block between the first transition up and the last transition down of the original FC-DenseNet network is removed to ASPP module. The ASPP module consists of 1 × 1 convolution and atrous sampling rates of 6, 12, and 18, respectively, and the output feature maps of these 4 atrous convolutions are combined with the output of the downsampling operation before. Finally, the concatenated feature map pass through another 1 × 1 convolution. At the same time, the original pooling layer is changed to convolution layer with 3 × 3 kernel size and stride of 2, in order to reduce the information loss in the max pooling operation. We use FC-DenseNet with 56 layers which has 4 layers per dense block and a growth rate of 12 as based network The proposed network (we name it ASPP-FC-DenseNet) finally has 4 transition down, 4 transition up and ASPP module, totally 47 layers, as shown in Figure 3.

In the high-resolution digital mammograms image, there is an extreme imbalance between foreground (tumor) and background classes, which causes the classifier to be more biased to the background class in training and leads to poor segmentation results. Therefore, we improve the simple softmax cross entropy loss function and take each class frequency of the image into consideration. Assume that the frequency of class *l* in the training data is *f*_*l*_, and the sum of the frequency of all categories (background and tumor) is 1; that is, ∑_*l*_*f*_*l*_=1. The inverse frequency of each class is added to the cross entropy loss function, effectively strengthening each pixel of less frequency classes.

(1) $$\begin{array}{c} {\text{loss}}_{\text{ce}}=-\frac{1}{N}\overset{N}{\underset{i=1}{\sum }}\frac{1}{f_{y_{i}}}y_{i}\text{ log }p_{i}, \end{array}$$

where *N* is the number of pixels of the image, *y*_*i*_ is the class label of pixel *i*, and *p*_*i*_ is the model's predicted probability for the pixel with the correct class label.

## 3. Results and Discussion

### 3.1. Data

**Figure 4:**
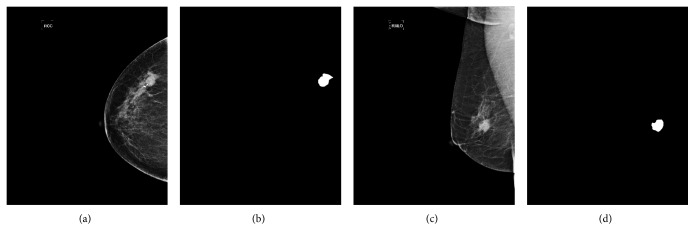
The mammogram data with different views and corresponding annotations. (a) CC view. (b) CC annotation. (c) MLO view. (d) MLO annotation.

The data used in our research are digital molybdenum target mammograms developed by the Department of Radiology in Henan Provincial People's Hospital. The mammograms dataset totally contains 190 patient cases, each of which contains the craniocaudal (CC) view and mediolateral oblique (MLO) view, as shown in Figure 4. That means there are 380 images in total. MLO and CC images are gray-level digitized mammograms with a resolution of 3328 (width) by 4096 (height) pixels saved as standard DICOM format. All the tumors in the mammograms were depicted by a professional radiologist in the hospital, as shown in Figure 4. We randomly divided the dataset into train set, validation set, and test set with no cross among them. The train set contained 230 images, and the validation and test set, respectively, contained 75 images.

### 3.2. Metrics

In this paper, we chose the Dice Index (DI) which is commonly used in most medical image segmentation task and pixel accuracy (PA) and Intersection Over Union (IOU) which is commonly preferred in natural image segmentation task to quantitatively evaluate the segmentation performance of the breast tumor segmentation algorithm. The calculation is shown as follows:

(2) $$\begin{array}{c} \text{DI}=\frac{2\times \text{TP}}{2\times \text{TP}+\text{FP}+\text{FN}},\\ \text{PA}=\frac{\text{TP}}{\text{TP}+\text{FN}},\\ \text{IOU}=\frac{\text{TP}}{\text{TP}+\text{FN}+\text{FP}}. \end{array}$$

Among them, TP refers to the number of pixels that are correctly divided into tumors. FP is the number of background pixels that are wrongly judged as the tumor. TN is the number of pixels that are correctly identified as the background, and FN represents the number of tumor pixels that are identified as background.

### 3.3. Experimental Evaluation and Discussion

We evaluated the proposed model on our collected mammogram dataset. The initial learning rate of our proposed network is set to 0.001, and the Adam optimization algorithm [41] with default beta values is used to update the gradient and network parameters. Dropout with a rate of 0.2 and batch normalization are also used as a regularizer. The training batch size is set to 1, and we train our model for 100 epochs to compensate for the smaller batch size. Every pixel value in the mammograms is normalized into 0-1 and subtracts the pixel mean value as the network input.

**Figure 5:**
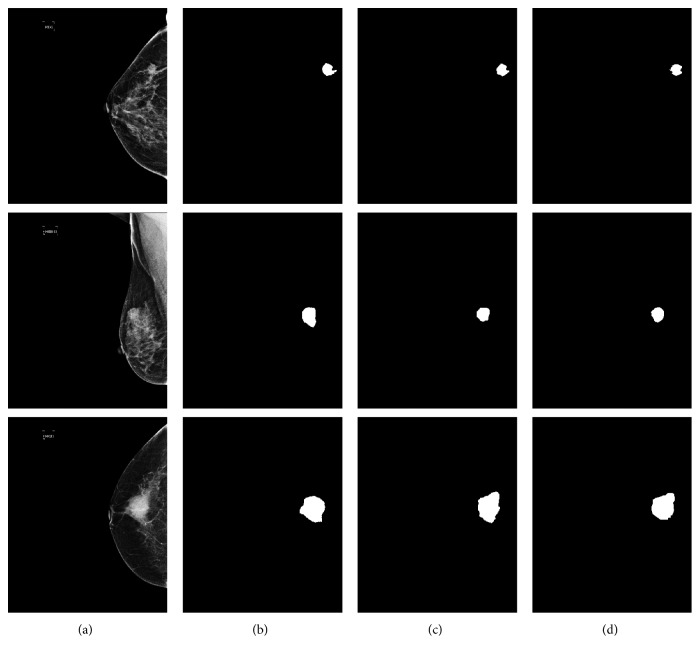
Segmentation results of different sizes of breast tumor. (a) Image. (b) FC-DenseNet. (c) ASPP-FC-DenseNet. (d) Ground Truth.

In order to verify the performance of the proposed ASPP-FC-DenseNet, we compared the ASPP-FC-DenseNet with the original FC-DenseNet containing 5 downsampling operations on the test set.  Figure 5 shows these two methods' segmentation results for different sizes of tumors. The ASPP-FC-DenseNet algorithm has a higher segmentation precision compared with FC-DenseNet and has obvious advantage on the edge preservation of different sizes of tumor. Therefore, the fusion of multiscale image information can help to obtain the multilevel image features and improve the performance of image segmentation which needs pixel level semantic recognition. At the same time, FC-DenseNet still has high recognition accuracy for the first two mammogram images with small difference in the internal and external gray values of tumor. It can identify the tumor location accurately and verify the effectiveness of FC-DenseNet for breast cancer segmentation.

As shown in Table 1, the mean Dice Index of ASPP-FC-DenseNet algorithm on the tumor segmentation test set is 0.7697, the mean IOU is 0.6041, and the mean pixel accuracy is 0.7983. Both the Dice Index and the IOU of ASPP-FC-DenseNet algorithm have a small increase. The Dice Index is increased by 3.42%, the IOU is increased by 1%, and the pixel accuracy is almost not improved. Because the pixel accuracy is mainly concerned with the false negatives rate of tumor pixels, and the Dice Index and the IOU consider the false negatives rate and misdetection rate of the tumor pixels at the same time, which can more comprehensively illustrate the segmentation precision of the algorithm. Therefore, it also reflects that ASPP-FC-DenseNet has a competitive advantage in reducing the misdiagnosis rate of the tumor pixels in the same case.

**Figure 6:**
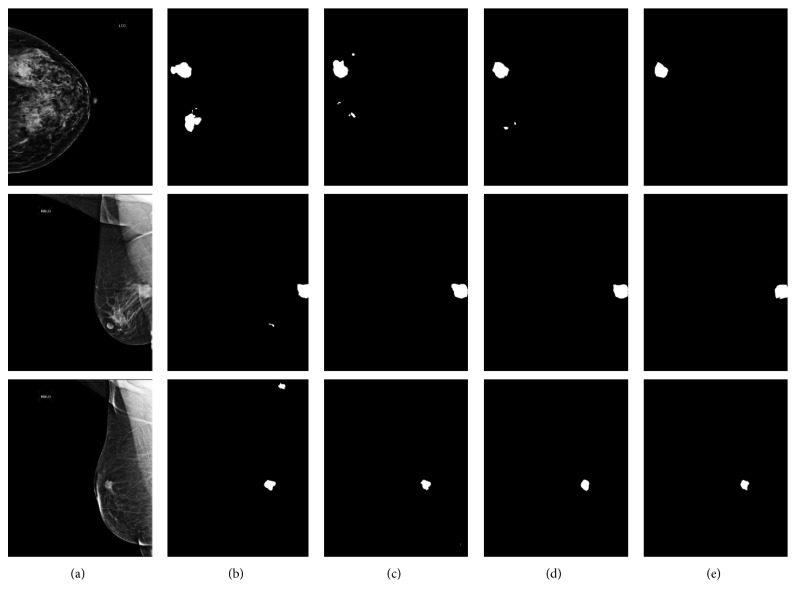
The tumor segmentation results of ASPP-FC-DenseNet model with different loss functions. (a) Image. (b) Dice loss. (c) No weighted loss. (d) Ours. (e) Ground Truth.

We proposed the weighted cross entropy loss to mitigate the extreme imbalance between foreground (tumor) and background pixels counts. We also compared with normal cross entropy loss (no weighted) and dice loss which is recently proposed in medical image segmentation [42] to show the importance of the weighted cross entropy loss. The tumor segmentation results of ASPP-FC-DenseNet model with different loss functions are shown in Figure 6. From the segmentation results, weighted loss model has a lower false negative rate, and the segmented tumor contour is more accurate than other two loss models. For this reason, the dice coefficient of weighted loss is obviously higher which is same with the quantitative comparisons of Table 2. This shows that the weighted cross entropy loss has a better performance in class imbalance problem compared with common cross entropy loss. The computation of dice loss might lead the gradient and training unstable, and sometimes influence the performance.

**Figure 7:**
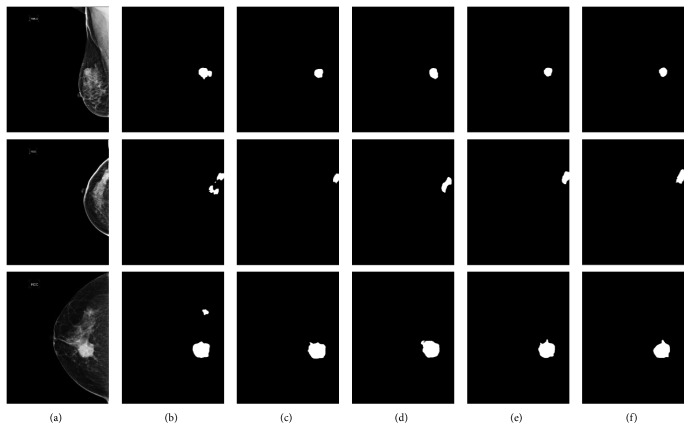
Tumor segmentation results of different CNNs. (a) Image. (b) U-Net. (c) PSPNet. (d) Deeplab v3+. (e) Ours. (f) Ground Truth.

We also performed experiments compared with the original PSPNet [43], deeplab v3+ [44], and U-Net to demonstrate the selected model superiority. The tumor segmentation results of different models are shown in Figure 7. Compared with other baseline models, our proposed ASPP-FC-DenseNet model has high segmentation precision for different size of tumors and complex backgrounds. All models have accurate tumor localization, but other three models could not obtain accurate tumor boundaries compared with our model. From these comparison results, we also found that U-Net had high false negative rate. Compared with U-Net, PSPNet and deeplab v3+ models all merge multiresolution image features and have a better segmentation performance. This also proves the importance of multiscale image features and verifies the advantage of added ASPP module at the same time.

The quantitative comparison results of these four models are shown in Table 3. Our model is obviously superior to the other three models on the three evaluation metrics. The minimal improvement also reaches 4%. Deeplab v3+ model with encode-decode structure and ASPP module has higher segmentation precision compared with the other two models, which also demonstrated the advantage of these two structures. But the decode module of deeplab v3+ model used simple bilinear upsample operation which might lose detailed low-level features. Our model refers to U-Net decode module to recover image resolution step by step and concatenates with image features in the encode module. Therefore, it has a higher segmentation precision.

We also select the level set [45], graph cut [46], and threshold segmentation algorithm for qualitative and quantitative comparison on the breast tumor segmentation test set.

**Figure 8:**
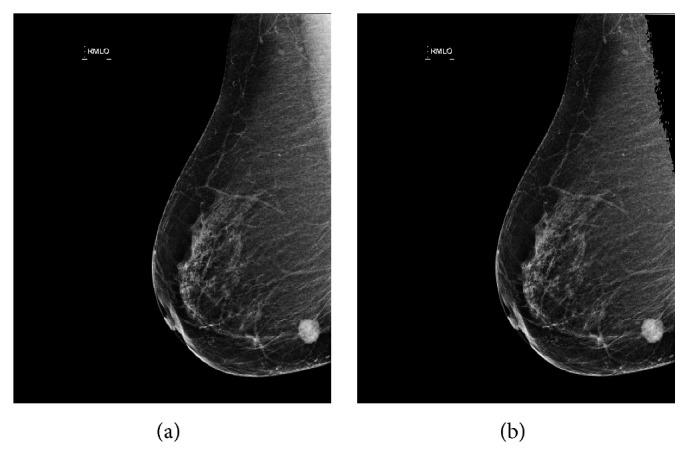
The original MLO mammogram and pectoralis deleted mammogram.

The gray value of the pectoralis in the MLO view image is very close to the tumor, which affects the segmentation precision. Before using the level set, graph cut, and threshold segmentation algorithm, the pectoralis of the MLO image is removed first according to the location information and the gray threshold, as shown in Figure 8. Threshold segmentation algorithm is a simple image segmentation algorithm. We used a double threshold segmentation method. Firstly, the initial tumor region is obtained by the iterative threshold segmentation algorithm. Then the final threshold segmentation result is obtained by calculating the gray mean value of the first step of the tumor region as the threshold of the second step segmentation. For the segmentation results of these three contrast algorithms, the area of connected region is calculated and the isolated small connected region is deleted as the final segmentation result.

**Figure 9:**
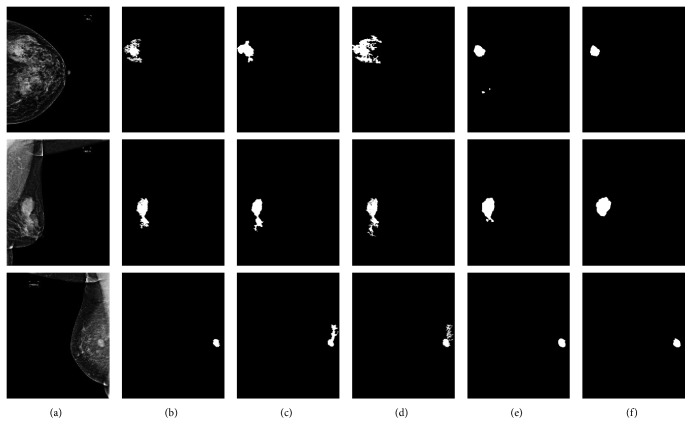
The tumor segmentation results compared with other segmentation algorithms. (a) Image. (b) Level set. (c) Grab cut. (d) Double threshold. (e) Ours. (f) Ground Truth.

The segmentation results of our proposed algorithm are compared with other three segmentation algorithms, as shown in Figure 9. It is obvious that other three segmentation algorithms using preprocessing that removes the pectoralis and postprocessing still have a poor segmentation performance compared with proposed ASPP-FC-DenseNet segmentation algorithm. The other three segmentation algorithms can accurately locate the location of the tumor, but it has a high misdetection rate, especially for the tumor images with small difference and similar characteristics inside and outside the tumor. This is due to the differences of the tumor grayscale, texture, and other characteristics with the normal breast tissue, so it is easy to locate the tumor position. Although level set and graph cut are interactive segmentation algorithms, which can minimize the energy function by manually setting the initial location of the tumor as a priori, they are mainly based on the tumor grayscale and texture properties to update the evolution curves and lack the high-level semantic information of the image. For the non‐tumor region whose characteristic is similar to tumor, these three algorithms have a poor segmentation result. The proposed algorithm has powerful feature extraction and representation ability and can obtain more accurate segmentation results.

The quantitative comparison results of these four segmentation algorithms are shown in Table 4. The proposed ASPP-FC-DenseNet algorithm has significant improvement on the three evaluation metrics compared with the other three segmentation algorithms. Compared with the graph cut algorithm, ASPP-FC-DenseNet improved 30.34% on the Dice Index, increased by 25.50% on the IOU, and increased by 17.63% on the pixel accuracy. Even compared to the level set which has better segmentation performance in the three algorithms, the proposed algorithm also increased by 17.08% on the Dice Index. The IOU increased by 11.48%, and the pixel accuracy increased by 11.70%. The segmentation precision was improved significantly. At the same time, it can be seen that the proposed algorithm is more effective in improving the Dice Index and IOU, indicating that under the same misdetection rate, the proposed algorithm has a lower false negative rate compared with the other three algorithms and has a higher segmentation precision.

## 4. Conclusions

In this paper, a fully convolutional network ASPP-FC-DenseNet, which combines multiscale image information, is proposed to achieve automatic segmentation of breast tumor. The algorithm uses FC-DenseNet which further exploits the feature reuse by using skip connections to help the upsampling path recover spatially detailed information from the downsampling path. Considering that five pooling layers in the network lead the small size tumor that cannot be identified easily, the number of pooling layers in the network is reduced to 4. Then the atrous spatial pyramid pooling module is added to the network after the last downsampling operation, which concatenates different field-of-views of image features through the combination of multiple sampling rates of atrous convolution. Finally, the loss function of the network is improved according to the proportion of the tumor pixels in the image, in order to weaken unbalanced class problem. Qualitative and quantitative experimental results prove that the algorithm proposed in this paper has high segmentation precision for various sizes and shapes of tumor mammograms without preprocessing and postprocessing and achieves automatic tumor segmentation.

## Figures and Tables

**Table 1:** The quantitative comparisons of the proposed and original FC-DenseNet algorithms.

Methods	DI	IOU	PA
FC-DenseNet	0.7355	0.5948	0.7968
ASPP-FC-DenseNet	0.7697	0.6041	0.7983

**Table 2:** The quantitative comparisons of ASPP-FC-DenseNet model with different loss functions.

Methods	DI	IOU	PA
No weighted loss	0.7151	0.5974	**0.8015**
Dice loss	0.7108	0.5920	0.7988
Ours	**0.7697**	**0.6041**	0.7983

**Table 3:** The quantitative comparisons of different CNNs.

Models	DI	IOU	PA
U-Net	0.6763	0.5608	0.7562
PSPNet	0.6785	0.5427	0.7202
Deeplab v3+	0.6827	0.5641	0.7072
ASPP-FC-DenseNet	0.7697	0.6041	0.7983

**Table 4:** The quantitative comparisons of the proposed model and other algorithms.

Methods	DI	IOU	PA
Level set	0.5989	0.4893	0.6813
Grab cut	0.4663	0.3491	0.6220
Threshold	0.5464	0.4322	0.6440
ASPP-FC-DenseNet	0.7697	0.6041	0.7983

## Data Availability

The digital mammogram data used to support the findings of this study have not been made available because of the patients' privacy.
